# Autosomal recessive hyper-IgE syndrome caused by *DOCK8* gene mutation with new clinical features: a case report

**DOI:** 10.1186/s12883-021-02324-3

**Published:** 2021-07-23

**Authors:** Jing Yang, Yan Liu

**Affiliations:** Tong Ji Hospital, Tong Ji Medical College, Huazhong University of Science and Technology, jiefang Ave. No. 1095, Wuhan, 430030 China

**Keywords:** Hyper IgE syndrome, *DOCK8* gene mutation, Facial paralysis, New clinic features

## Abstract

**Background:**

Autosomal recessive hyper-IgE syndrome (AR-HIES) caused by *DOCK8* gene is a rare immunodeficiency disease, the main clinical manifestations include recurrent Eczema-like rash, skin and lung abscesses, accompanied with increased serum IgE level. Here, we report a 7-year-old Chinese girl with a new clinic features caused by *DOCK8* gene mutations.

**Case presentation:**

A 7-year-old girl was admitted to our hospital because of abnormal walking posture. The clinical manifestations of the patient included abnormal gait, eczema-like rash, fingertip abscess, high muscle tone, and facial paralysis. Among them, high muscle tone and facial paralysis are new clinic features which have not been reported previously. The blood eosinophils and serum IgE levels were significantly increased, and the lymphocyte subsets indicated a decrease of T lymphocytes. The magnetic resonance imaging (MRI) of her brain suggested myelin dysplasia and brain atrophy. Two novel compound heterozygous mutations (c.1868 + 2 T > C and c.5962-2A > G) of *DOCK8* gene were identified by whole exome sequencing. By literature review, there are 11 mutations of *DOCK8* gene in Chinese AR-HIES patients.

**Conclusions:**

Two novel splice-site mutations(c.1868 + 2 T > C and c.5962-2A > G) of *DOCK8* gene and new clinic features were found in a Chinese girl with AR-HIES, which extends our understanding of *DOCK8* gene mutation spectrum and phenotype of AR-HIES in children.

## Background

Hyper IgE Syndrome (HIES), also known as Job Syndrome, was first reported by Davis in 1966 [1], HIES is a rare immunodeficiency disease, the main clinical manifestations include recurrent Eczema-like rash, skin and lung abscesses, accompanied with increased serum IgE levels. The incidence of this disease is less than 1/100,000, and it usually occurs in infants and young children without gender and ethnic differences [2]. HIES is divided into autosomal dominant HIES (AD-HIES) and autosomal recessive HIES (AR-HIES) according to different inheritance gene. AD-HIES caused by *STAT3* mutation and AR- HIES caused by *PGM3*, *SPINK5*, *DOCK8* and *TKY2* mutations have been reported [3].

The *DOCK8* gene was firstly reported by Englharts et al. in 2009 [4], it contains 46–48 exons (transcript: NM_001190458.1 at chr9:273,048–465,259; NM_001193536.1 at chr9:273,048–465,259) spanning 190 kb, and maps chromosome 9p24.3. DOCK8 is a member of the DOCK180 family of atypical guanine nucleotide exchange factors (GEF). The DOCK180 family protein domain is also called DHR, binding domain or CZH binding domain, it can combine with RAC, CDC42 and other families to form a complex structure, remove GDP, and promote GTP to bind and active PhoGTP enzyme. However, the substrate that binds to the DOCK8 molecule is currently unclear [5]. DOCK protein plays an important role in cytoskeletal organization, affecting the migration of dendritic cells. DOCK8 deficiency leads to the continuous existence of germinal center B cells, early T cell apoptosis, and reduces natural killer cell (NK cell) toxicity [5]. The pathogenesis of AR-HIE patients caused by DOCK8 gene mutation is related to cellular and/or humoral immunity. The number of T cells is significantly lower than normal, and may be accompanied by a decrease in memory B cells.

## Case presentation

**Fig. 1:**
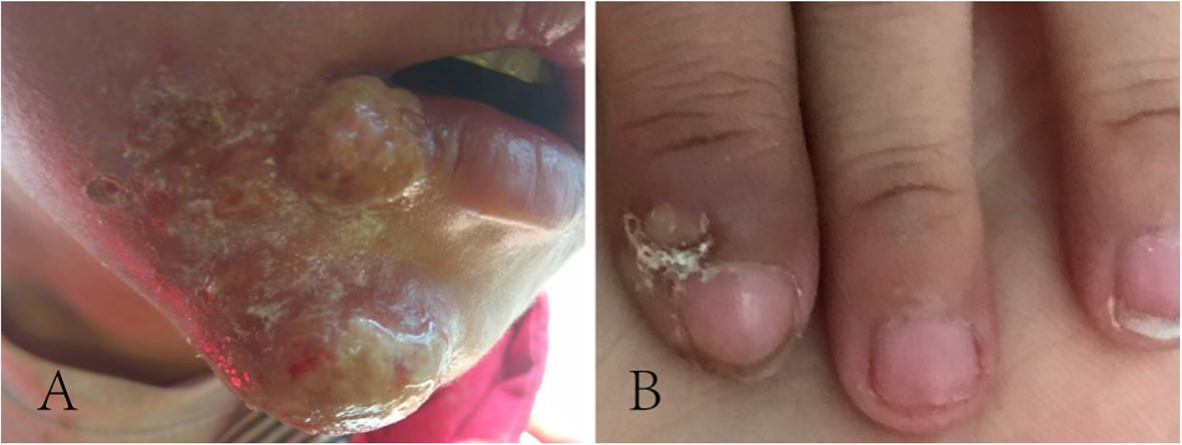
**A **The patient with repeated eczema-like rash on the face. **B **The patient with repeated suppuration of the fingertips

**Fig. 2:**
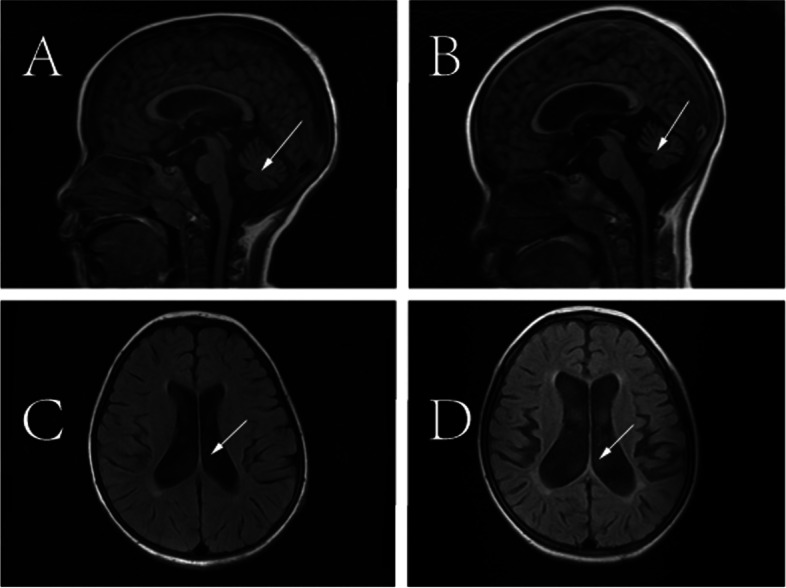
Comparison of MRI image between the first admission and recent time. **A** and **C** MRI on the first admission of the patient. **B** and **D** Recent MRI of the patient

**Fig. 3:**
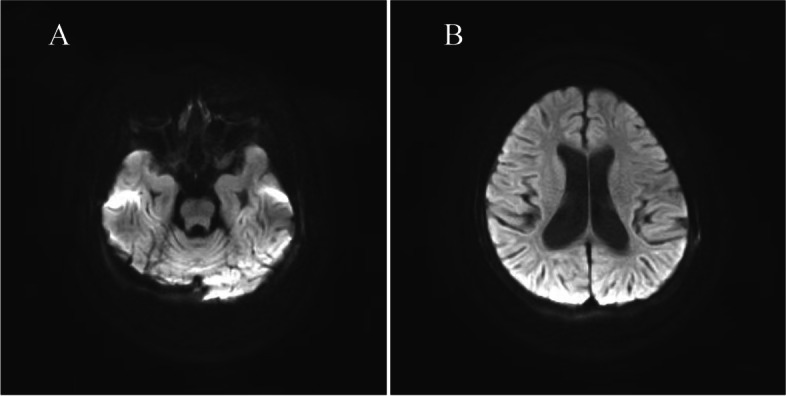
The heard MRI-DWI of the patient

The index patient was a 7-year-old girl from a non-consanguineous family, and she was admitted to our hospital with abnormal walking posture. Her growth and development assessment indicated height of 107 cm(< 3rd) and weight of 20 kg(10th-25th). The patient was able to walk normally at 1 year old, one year later, she appeared abnormal gait, and was prone to wrestling. Therefore, she was misdiagnosed as cerebral palsy, and her symptoms did not improve significantly after rehabilitation. When she was 3 years old, she showed repeated facial eczema, suppuration, and finger abscesses (see Fig. 1 A, B). Physical examination showed eczema-like rashes and abscesses on the face, and the fat under the skin was thin. Her muscle tension of both lower limbs was high, and tension of ankle joint was high. Muscle strength was normal, ankle clonus was positive. Foot inversion, knee extension, and gait instability were observed when walking. Since last year, the patient had 6 times unilateral peripheral facial paralysis, which is a clinic feature not seen in previous reports. Her parents had no clinical symptom as same as the patient. This patient's blood routine results suggested elevated eosinophils, the serum IgE was more than 3200 IU/ml (≤ 90 IU/ml). The results of serum lymphocyte subsets suggested that total T lymphocytes were low and total B lymphocytes were normal; helper T cell/suppressor T cell (Th/Ts) fluctuated between 0.21 and 0.42, which were significantly lower than normal levels (1.03–2.09); the percentage of assisted/induced T lymphocytes fluctuated between 9.26%-14.75%, which were lower than the normal level (18.2%-30.6%); the NK cells ranged from 18.01% to 31.79%, which was higher than the normal level (6.9%-19.3%). Compared with the first admission 2 years ago, the recent MRI of patient suggested that myelin dysplasia and the cerebellar atrophy was aggravated (see Fig. 2), the MRI-DWI of her head was shown in Fig. 3.

**Fig. 4:**
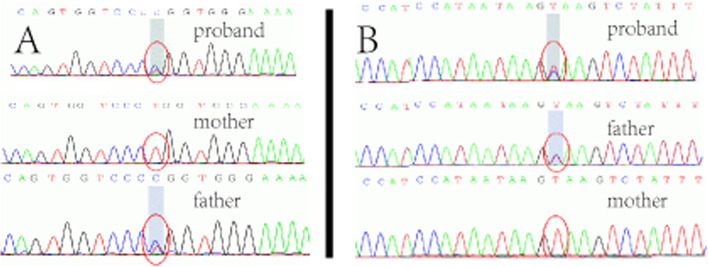
Sanger sequencing results of the patient and her parents. **A** chr9:370,302, c.1868 + 2 T > C **B** chr9:452,009, c.5962-2A > G

There were two splice-site mutations (c.1868 + 2 T > C, c.5962-2A > G) of DOCK8 gene in the patients’ WES (see Fig. 4). His father and mother carried the same heterozygous mutation as the patient, respectively, which were consistent with compound heterozygous mutation. According to ACMG criteria, the two splice-site mutations were classified as possibly pathogenic (PVS1 and PM2).

### Literature review of Chinese patients with *DOCK8* gene mutation

To date, more than 130 mutations have been reported in *DOCK8* gene all over the world, gross deletions are the most mutations among them. There are 11 different mutations of *DOCK8* gene in Chinese patients with autosomal recessive high IgE syndrome (Table 1) [6–11]. Among the 11 patients, 3 patients had atopic dermatitis, 8 patients had eczema, 10 patients had a certain degree of infection and allergy symptoms. Among these 10 infected patients, 3 patients had repeated respiratory infection, 3 patients had sinusitis, 8 patients had otitis media, 5 patients had stomatitis, 2 patients had conjunctivitis. For pathogens, including herpes simplex virus (HSV), human papilloma virus (HPV), cytomegalovirus, herpes simplex virus, measles virus, candida, hepatitis B virus. It is worth noting that none of the 11 patients had neurological symptoms. Now we found a compound heterozygous mutation (c.1868 + 2 T > C, c.5962-2A > G) of *DOCK8* gene, which was not reported in previous Chinese cases, in addition, the patient had neurological symptoms.

**Table 1:** Summary of Chinese patients with DOCK8 mutation

Index Case	Sex	Mutation	Clinic feature			
			Infection	Allergy	Atopic dermatitis	Eczema
**1**	F	c.1126_1285del	Yes	Yes	Yes	No
**2**	M	c.646_647del	Yes	Yes	Yes	No
**3**	M	c.646_647del	No	No	Yes	No
**4**	F	c.5842delG, c.5843C > A	Yes	Yes	No	Yes
**5**	M	Exon11 hom del Exon 12–33 het del	Yes	Yes	No	Yes
**6**	F	c.3152delG	Yes	Yes	No	Yes
**7**	F	c.3152delG	Yes	Yes	No	Yes
**8**	F	Exon 2 hom del Exon 1, 3–39 het del	Yes	Yes	No	Yes
**9**	F	Exon 7 hom del Exon 8–10 het del	Yes	Yes	No	Yes
**10**	M	c.1278–1279 del TG	Yes	Yes	No	Yes
**11**	F	c.4886 G > A	Yes	No	No	Yes

*F* female, *M* male

## Discussion and conclusions

The present paper has reported a compound heterozygous mutation (c.1868 + 2 T > C, c.5962-2A > G) of *DOCK8* gene in a Chinese family with AR-HIES. The two splice-site mutations and facial paralysis were not reported previously in the patients with *DOCK8* gene mutation. Combined with her clinical features, laboratory test results, and next-genetic sequencing results, we consider that she can diagnose high IgE syndrome. Her lower extremity muscle tone became more and more hypertensive, and cerebellar atrophy also worsened. Since only DOCK8 gene mutations were found in WES and CNV, we thought that her new clinic features were caused by DOCK8 gene mutations. At the beginning of the patients’ admission, we have excluded cerebrovascular, in addition, her symptoms could not be explained by other causes other than the DOCK8 gene mutation.

The characteristics of AR-HIES patients caused by *DOCK8* gene mutation include the significant increase of serum IgE level and eosinophilia in almost all patients. Serum IgG levels are usually normal or increased, IgM levels are usually reduced or decreased with age, and IgA levels can be increased, decreased, normal [12]. Zhang et al. suggested 90% of patients had lower total T cells and total B cells, all patients had lower CD4 + T cells, 36% had lower B cells, and 60% had lower NK cells in 2009 [13]. For the patient, her serum IgE was more than 3200 IU/ml, eosinophil count increased, the total T cell count was low, and the total B cell count was normal. Common clinical features of AR-HIE caused by DOCK8 gene mutation include allergy, infection, malignant tumors. The patients caused by DOCK8 gene mutation are susceptible to viral infection, which is related to cellular and humoral immunity. On the one hand, memory B cells are greatly reduced, which makes patients to virus infection. On the other hand, the patients may have excessive lymphocyte proliferation and abnormal IgE plasma cell proliferation. The number of T cells of the patients is significantly lower than normal. Defective T cells are related to cellular immunity and easily lead to viral infections in patients with DOCK8 deficiency. The main viral infection etiologies in patients with DOCK8 deficiency include herpes simplex virus, varicella-zoster virus, human papilloma virus and molluscum contagiosum virus.

In addition, a small number of patients may have severe central nervous system lesions, such as hemiplegia, seizures, ischemic infarction, and subarachnoid hemorrhage [6], central nervous system vasculitis combined with stroke [7]. Among the 20 families reported by Engelhardt et al. [14], 10 patients had neurological symptoms, including 4 meningitis, 2 CNS vasculitis, 2 severe neurological disease (PML), and 2 fatal encephalitis. For the novel clinic phenotype of facial paralysis, we considered that it is caused by autoimmune deficiency. In a clinical study of Bell’s palsy in adults, Aviel et al. suggested that there are some alterations in the lymphocyte subsets of the peripheral blood during the acute stage of the disease [15]. Gorodezky et al. suggested that compared with control patients, total T cells (CD3) and T helper/inducer cells (CD4) have been decreased in the acute phase of the disease [16].

Compared with AD-HIES, AR-HIES has no clear diagnostic criteria, according to the clinical characteristics and laboratory tests of patients, genetic testing as early as possible is an important means to diagnose such disease.

The patient was identified two heterozygous mutations in the spite-site of the *DOCK8* gene (c.1868 + 2 T > C, c.5962-2A > G). His father and mother carried the same heterozygous mutation as the patient, respectively, which were consistent with autosomal recessive inheritance. The mutations of the patient are located in the region of the splice site, according to the ACMG guidelines, the two mutations are satisfied as PVS1 + PM2. Splice-site mutation may affect the expression of *DOCK8* gene and the synthesis of DOCK8 protein, combined with the clinical features, it is considered as pathogenic mutations. There are 11 Chinese patients with AR-HIES caused by *DOCK8* mutations in our study. Among them, 10 had large deletions in the exon region, which consistent with common mutation types in HGMD database. There are splicing-site mutations in the DOCK8 gene reported abroad, but there are differences in the clinical phenotype of our patient. Engelhardt et.al reported a patient with splicing-site mutation of DOCK8 gene, her neurological symptoms were meningitis [17]. Patients with AR-HIES caused by *DOCK8* gene mutation can be treated with gamma globulin to reduce the incidence of infection, corresponding antiviral treatment can be given to patients with virus infection. Allogeneic hematopoietic stem cell transplantation has achieved relatively definite therapeutic effects in such diseases, and there have been case reports of successful allogeneic hematopoietic stem cell transplantation [18]. In a cohort of 136 people reported by Susanne et al., a study of the overall survival probability of allogeneic hematopoietic stem cell transplantation showed that: 87% at 10 years old, 47% at 20 years old, and 33% at 30 years old [19].

In summary, we report a novel clinical phenotype of the AR-HIES patient caused by *DOCK8* gene mutation. Two novel mutations (c.1868 + 2 T > C, c.5962-2A > G) of *DOCK8* gene mutation was found in the Chinese patient, which extends our understanding of *DOCK8* gene mutation spectrum and phenotype of AR-HIES in children.

## Data Availability

All data generated or analysed during the current study are included in this published article.

## Abbreviations

AR-HIES: Autosomal recessive high IgE syndrome
MRI: Magnetic resonance imaging
HIES: High IgE Syndrome
AD-HIES: Autosomal dominant high IgE syndrome
HSV: Herpes simplex virus
HPV: Human papilloma virus
